# Evidence for Multi-Organ Infection During Experimental Meningococcal Sepsis due to ST-11 Isolates in Human Transferrin-Transgenic Mice

**DOI:** 10.3390/microorganisms8101456

**Published:** 2020-09-23

**Authors:** Michael Levy, Myriam Aouiti Trabelsi, Muhamed-Kheir Taha

**Affiliations:** 1 Institut Pasteur, Invasive Bacterial Infection Unit, 28 rue du Dr Roux, 75724 Paris, France; myriam.atrabelsi@gmail.com (M.A.T.); muhamed-kheir.taha@pasteur.fr (M.-K.T.); 2 Paediatric Intensive Care Unit, Robert-Debré University Hospital, Assistance Publique Hôpitaux de Paris, 75019 Paris, France; 3 Université de Paris, 75019 Paris, France

**Keywords:** Neisseria meningitidis, septicaemia, animal model, mouse, purpura

## Abstract

The description of invasive meningococcal disease that is provoked by *Neisseria meningitidis* (Nm) is frequently restricted to meningitis. However, a wide panel of clinical presentations can be encountered including severe forms with intense inflammatory reaction leading to multi-organ failure. Several human factors are involved in the development of invasive infections such as transferrin, factor H or CEACAM1. In this study, we used an experimental meningococcal infection in transgenic mice expressing the human transferrin to show multi-organ infection. Mice were infected by an intraperitoneal injection of bacterial suspension (1.5 × 10^7^ colony-forming unit/mouse) of a bioluminescent serogroup C strain belonging to the clonal complex ST-11. Dynamic imaging and histological analysis were performed. The results showed invasion of tissues by Nm with bacteria observed, outside blood vessels, in the kidneys, the heart and the brain as well as skin involvement. These data further support the systemic aspect of invasive meningococcal disease with involvement of several organs including skin as in humans. Thus, our model can be used to study severe forms of meningococcal invasive infections with multi-organ failure.

## 1. Introduction

*Neisseria meningitidis* (Nm) is a strict human pathogen that is frequently carried asymptomatically in the nasopharynx. Nm can also provoke invasive meningococcal disease (IMD) including meningitis and septicaemia that may lead, in its severe forms, to *purpura fulminans* (PF) [1]. The disease is due to the ability of particular meningococcal strains to cross the epithelial barriers to invade the bloodstream. Nm can then spread, proliferate rapidly and provoke an intense inflammatory response that can lead to PF, a septic shock with skin haemorrhagic rash and disseminated intravascular coagulation, evolving to multi-organ failure [1]. Studies using molecular methods cluster bacterial isolates genotypes called clonal complexes (cc) and meningococcal invasive isolates responsible for IMD frequently belong to few hyper-invasive cc [2]. Hyper-invasive isolates, such as the cc11 isolates, have a remarkably high association with IMD and are consistently associated with apoptosis induction, inflammatory reaction and death [3,4]. The negative outcome of IMD has been linked to a systemic coagulation dysregulation, triggered by monocytes overexpressing active tissue factor, as well as to the excessive inflammatory reaction [5,6]. Recent works have also shown that, through vascular invasion, Nm could colonize the human coronary vasculature and induce myocarditis [7]. However, while the tropism of Nm for blood vessels seems to be well established, the multi-organ invasion has been suggested during meningococcal sepsis [6] but still needs direct evidences. Relevant animal models may be helpful to address this point. Transgenic mouse models for meningococcal disease have been developed and used to study pathophysiological aspects of invasive meningococcal infections using intraperitoneal or intranasal experimental infection. These models used human CEACAM1 transgenic mice [8,9], human CD46 transgenic mice [10,11] and human complement factor H transgenic mice [12,13]. However, bacterial growth in these models is not efficient. In fact, Nm specifically uses human iron sources to grow and spread [14,15]. Other models also exist such as those using human engraftment that allows addressing vascular colonization [16,17,18,19]. Transgenic mice expressing the human transferrin provide Nm with an appropriate human iron source; the human transferrin. This model has been used to study several aspects of meningococcal virulence. We aimed in this work to address the multi-organ feature of IMD using transgenic mice expressing the human transferrin.

## 2. Materials and Methods 

### 2.1. Bacterial Strain

We used the meningococcal strain 24198LUX that is derived from the strain 24198 (serogroup C strain that belongs to ST-11 clonal complex, cc11) by the insertion of the luciferase operon (luxCDABE) on the chromosome under the control of a meningococcal promoter of *porB* gene. Bacteria were grown on Gonococcal base (GCB) medium supplemented with Kellogg supplements. The bioluminescent signal is an ATP-dependent process that requires viable bacteria with no signal detected in non-infected mice as previously reported [20]. Correlation between colonies forming units (CFU) and bioluminescent signals were evaluated by measuring signal from serial bacterial dilutions in a black ELISA plate. 

### 2.2. Experimental Infection in Mice 

Experimental meningococcal infections were performed in 8 week-old female BALB/c transgenic mice expressing human transferrin [21] by intraperitoneal injection of 1.5 × 10^7^ colony-forming unit (CFU)/mouse diluted in 0.5 mL of saline. This dose was selected to insure consistent infection in all mice [20]. 

### 2.3. Dynamic In Vivo Imaging

After 24 h of infection, a group of 5 mice were examined for skin aspects and three-dimensional (3D) imaging of mice was first performed by an IVIS Spectrum CT Xenogen Corp., Alameda, CA, USA). The mice were euthanized after bioluminescence by intraperitoneal injection of an overdosed mixture of xylazine and ketamine (MERIAL, Villeurbanne, France). This method combines an X-ray image with bioluminescence CT scan acquisitions overlaid with colour representations of luminescence intensity, measured in total photons/sec.

On another set of experiments, a group of 5 mice were euthanized after six hours of infection and several organs were extracted (brain, liver, spleen and kidney) and were analysed for two-dimensional bioluminescence using an IVIS 100 system and Living Image v. 4.3.1 software (Xenogen Corp., Alameda, CA, USA) as previously described [21]. Unlike IVIS Spectrum CT bioluminescent imaging, this method (using IVIS 100 system) allows rapid and almost immediate testing of organs and hence detecting reliable signals that decrease rapidly after animal death. All experiments were performed in duplicates. Each type of experiment was repeated twice.

### 2.4. Histological Analysis

Histological analyses were performed on extracted organs (heart, brain, kidney, skin) after 24 h of infection and after buffer perfusion. For the skin lesion, we checked for the presence of haemorrhagic lesions in the ears as they have little no furry aspect and therefore easy to watch without intervention.

The organs were fixed in 4% buffered formalin and embedded in paraffin. Sections of 3 µm were performed and stained using the haematoxylin and eosin (H&E) method and Gram staining was also performed using a Kit according to the recommendations of the manufacturer (Conda Laboratories, Madrid, Spain).

### 2.5. Ethical Aspects

This study was carried out in strict accordance with the European Union Directive 2010/63/EU (and its revision 86/609/EEC) on the protection of animals used for scientific purposes and under the administrative authorization for animal experimentation (Permit Number 75-1554). The protocol was approved by the Institut Pasteur Review Board that is part of the Regional Committee of Ethics of Animal Experiments of Paris Region (Protocol 99-174). 

### 2.6. Statistical Analysis

Correlation between CFU and bioluminescence measures were analysed by linear regression using GraphPad InStat version 3.06 (GraphPad Software, San Diego, CA, USA).

## 3. Results

**Figure 1:**
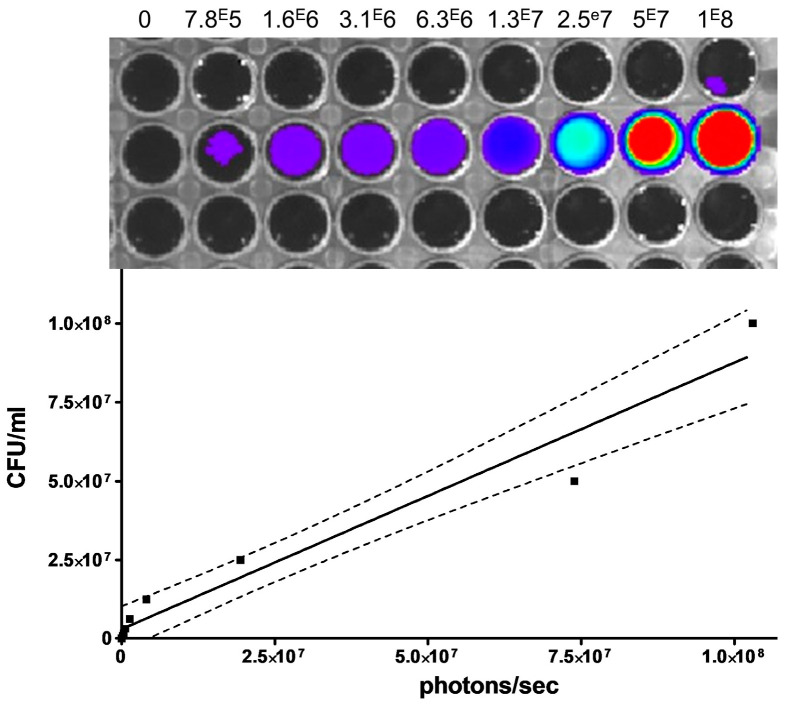
A serial of two-fold bacterial dilutions of the meningococcal bioluminescent strain 24198 were performed in the wells of a black 96-wells plate (from 1 × 10^8^ to 7.8 × 10^5^ CFU/mL) as indicated on the scales above the wells. Luminescence intensity was then measured and expressed as total photons/sec. The correlation between CFU and bioluminescent signals was analysed by linear regression (*r*^2^ = 0.95) and Spearman test (*p* < 0.0001). The curve (solid line) is shown with the 95% confidence interval (dashed lines).

As depicted in Figure 1, there was a consistent correlation between bacterial CFU and the bioluminescent signal. The same correlation was found between inoculated bacterial CFU and in vivo bioluminescence in this mice model [21,22].

**Figure 2:**
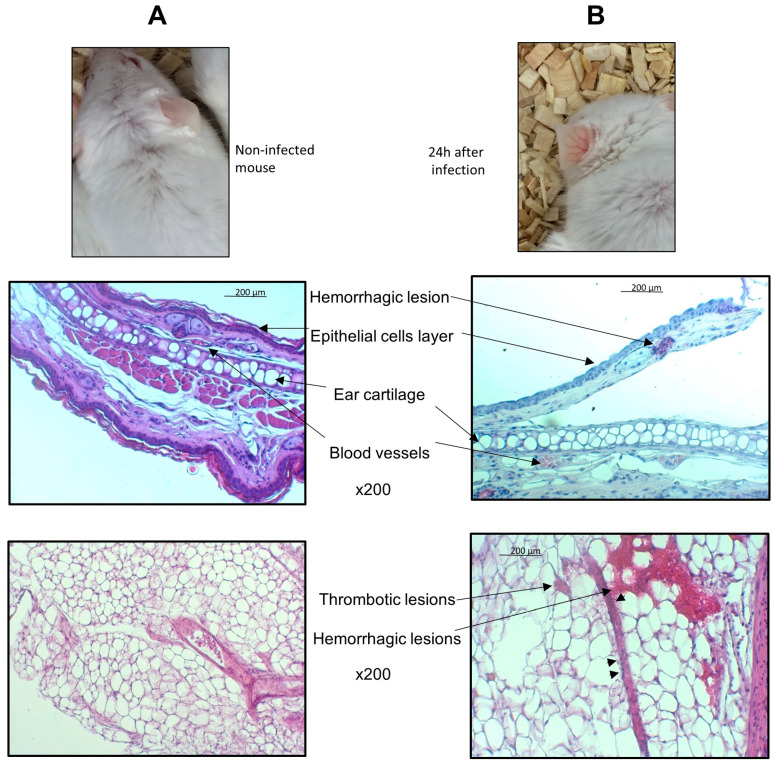
Transgenic BALB/c mice expressing human transferrin were infected by intraperitoneal injection of 1.5 × 10^7^ CFU of the bioluminescent *N. meningitidis* strain 24198LUX. Mice were examined before (**A** top) and after 24 h of infection (**B** top). Mice before infection showed no skin features but had signs of vasculitis after 24 h of infection that could be seen on the ears. Skin samples of the ear were collected in mice before infection (**A** middle) and after 24 h of infections (**B** middle) and were embedded in paraffin. Microscope examination at magnification ×200 are shown for H&E staining of 3 µm sections of paraffin embedded organ. The ear skin section of infected mice (**B** middle) showed haemorrhagic lesions outsides blood vessels. A 3 µm H&E stained section at magnification ×400 with of paraffin embedded omentum (fold of visceral peritoneum) with a longitudinal blood vessel in the filed showing signs of vasculitis (**B** bottom compared to **A** bottom). Note the haemorrhages around the vessel and endothelialisation (arrow heads) of leucocytes at the periphery of the vessels.

After 24 h of infections, mice had signs of clinical infection as previously published [22] and skin features that could easily be seen on the ears with macroscopic vasculitis aspect. These lesions were also observed at the site of infection where a section of the omentum showed haemorrhagic and thrombotic lesions of blood vessels with endothelisation of leucocytes at the periphery of the vessels (Figure 2).

**Figure 3:**
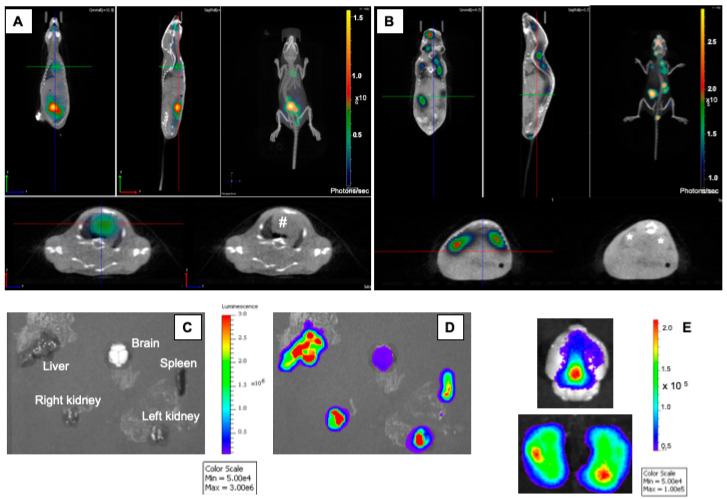
Transgenic BALB/c mice expressing human transferrin were infected by intraperitoneal injection of 1.5 × 10^7^ CFU of the bioluminescent *N. meningitidis* strain 24198LUX. A 2D CT Scan coupled with bioluminescence imaging was performed (**A** and **B**). Images depict CT scan acquisitions overlaid with colour representations of luminescence intensity, measured in total photons/sec and indicated on the scales. The CT scan was performed from a ventral (**A**) and from a dorsal view (**B**). The heart is highlighted by (#) and the kidneys are highlighted by (*) in the image without bioluminescence. In a distinct experiment, the analysis for bioluminescence was performed using an IVIS 100 system (Xenogen Corp., Alameda, CA, USA). Organs were withdrawn and are indicated in the image without bioluminescence (**C**). Bioluminescent imaging of withdrawn organs showed a high number of bacteria in the kidneys, the liver, the spleen and the brain (**D**). When focusing on the brain (**E**), the signal was diffuse as it is shown on the dorsal view (**E** upper part) and localized within the ventricular space when both hemispheres were imaged (**E** lower part).

The mice were imaged for three-dimensional (3D) imaging (computerized tomography scan) after 24 h of infection to detect bioluminescent signals. Bioluminescent signals were detected in the heart (Figure 3A, ventral view) and in the kidneys (Figure 3B, dorsal view).

The multi-organ involvement was further confirmed in another experiment of bioluminescent imaging on extracted organs after six hours of infection. Bioluminescent bacteria were also detected in the brain, the kidneys, the liver and the spleen (Figure 3C–E). 

**Figure 4:**
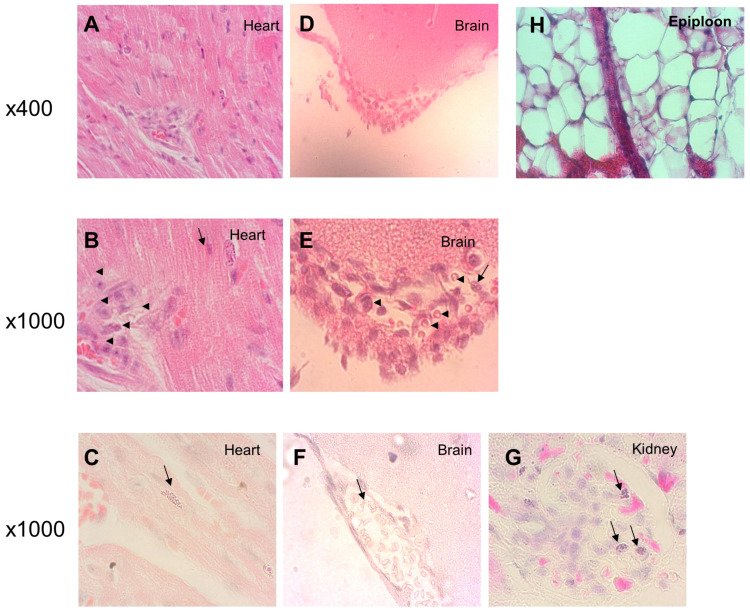
Transgenic BALB/c mice expressing human transferrin were infected by intraperitoneal injection of 1.5 × 10^7^ CFU of the *N. meningitidis* (Nm) bioluminescent strain 24198LUX. Organs were then withdrawn after 24 h of infections and were embedded in paraffin. Microscope examination at magnification ×400 and ×1000 are shown for H&E staining of 3 µm sections of paraffin embedded organs and revealed visible Nm (arrow) among the heart muscle fibres (**A,B**) and in the meninges (**D,E**) with inflammatory cells indicated by arrowheads (**B,E**). **C**, **F** and **G** respectively showed Nm (arrows) revealed by Gram staining in the heart, the meninges and the kidney of infected mice.

Histological analyses performed on extracted organs after 24 h of infection revealed visible Nm. Figure 4 shows examples of these stained sections with meningococcal localization between muscular fibres in the heart (Figure 4A–C), in the meninges (Figure 4D–F) and in the kidney (Figure 4G) outside the blood vessels and capillary.

Inflammatory reaction is observed by the presence of inflammatory cells in the meningeal space and in the heart (Figure 4B,E) and by haemorrhagic lesions of the skin (Figure 2B).

## 4. Discussion

Nm specifically requires human iron sources for growth. The human transferrin provides this source when it is produced in transgenic mice or if injected intraperitoneally in non-transgenic mice [15]. While other models may only insure meningococcal adhesion [8], the bacterial growth in transgenic mice expressing the human transferrin renders the model reliable for invasive meningococcal infections and enable several applications using several routes of infection (intraperitoneal and intranasal) [21]. We have extensively used this model to analyse several biological aspects of meningococcal infection. For example, the impact of reduced susceptibility to penicillin on the effectiveness of antibiotic treatment [23] as well as the impact of corticosteroids as an adjuvant treatment [22,24] could be studied. The expression of the human transferrin did not provoke any immunodeficiency in transgenic mice and leukocyte counts (neutrophils, lymphocytes and monocytes) did not differ significantly from those in wild type mice [21]. Using bioluminescent bacteria adds an additional advantage to the model as it allows following the bacteria spread in real-time. This spread is expected to reflect the blood flow rate in different organs and the differential rate of bacterial growth that may differ. We have recently reported that an isogenic mutant that are unable to use haemoglobin, as an iron source may be less effective in spreading into the spleen, a frontline organ in mechanical filtration of red blood cells and in recycling haemoglobin [19]. In the current work, we provided new evidence on the systemic aspect of the experimental infection in transgenic mice in parallel with the early stages of invasive infection in humans. Bacterial tropism to different tissues and organs may be important to explain the different signals in different organs. 

In this work, we used experimental meningococcal infection in transgenic mice expressing the human transferrin and showed that infection with hypervirulent strains of Nm such as those belonging to the cc11 led to high multi-organ infection in the kidneys, liver, spleen, heart and brain. These results are in accordance with the results obtained on porcine model of Nm sepsis that also demonstrated a major influx of bacteria in multiple organs like kidney, lungs, liver and spleen [6] and challenge the view of meningococcal sepsis as being an exclusive intravascular infection. This rapid systemic spread of meningococci is also associated with an inflammatory response as shown in the meningeal space and the heart (Figure 4B,D,E) and as we previously shown in the liver with multiple foci of hepatocellular necrosis, neutrophilic infiltration and occasional intravascular thrombi [22]. In addition, we demonstrated vasculitis in skin and in the omentum with haemorrhagic and thrombotic lesions that are usually seen in human disease [1] but rarely observed in animal models. However, low numbers of bacteria are observed in the involved organs. This may be due to Nm remains mainly in blood vessels (vasculitis) and due to less efficient interaction between Nm and murine endothelial cells of blood vessels to allow bacteria crossing the vessels to invade tissues [7]. The inflammatory response provoked by Nm and its endotoxin during vasculitis may explain early atypical presentation of IMD such as predominant abdominal pain [25]. Recently, a meningococcal duodenitis was also reported and further highlights the systemic hematogenous spread of meningococci and that IMD is not solely meningitis [26]. This multi-organ meningococcal infection is also responsible for the extra-meningeal forms of IMD such as arthritis and pericarditis. 

Our data help further understanding the systemic spread of IMD and increase the awareness of atypical forms and manifestations of this rapid invasive infection.
